# Supplementary material for: Communities of Digger Wasps (Hymenoptera: Spheciformes) along a Tree Cover Gradient in the Cultural Landscape of River Valleys in Poland
Source: Insects. 2024 Jan 29;15(2):88. doi: 10.3390/insects15020088 (PMC10889137; doi:10.3390/insects15020088)
Supplement: Supplementary file 1 [file insects-15-00088-s001.zip › insects-2713350-supplementary.pdf]

insects

MDPI

Supplementary Materials

Communities of Digger Wasps (Hymenoptera: Spheciformes) along a Tree Cover Gradient in the Cultural Landscape of River Valleys in Poland

Piotr Olszewski, Tim Sparks, Lucyna Twerd and Bogdan Wiśniowski

Table S1. Numbers, estimated flight period, information on adult foodplants, larval prey, nesting type and prey types of the species recorded at nine sites in the cultural landscape of the river valleys of northern Poland during 2011–2013; *n* – number of samples.

No	Species and status (where appropriate)	Sites									Total number of specimens	Observations from current research		Data from Literature	
		**PT** *n* = 82	**SI** n = 96	**GR** *n* = 68	**ZP** *n*=70	**GN** *n*=87	**TP** *n*=67	**TG** *n*=73	**GE** *n*=58	**EL** *n*=74	**PT-EL**	**Flight period**	**Remarks on prey (P) and food plants of adults (F)**	**Nesting**[3,4]	**Prey**[1,2,?]
1	*Alysson spinosus* (Panzer, 1801)		24	32		4		1	1		62	June – early August	(P) Delphacidae and Cicadellidae (*Jassargus* spp. and *Streptanus* spp.) (F) honeydew on the leaves of *Tilia cordata* Mill.	En[3]	He-ot[1]
2	*Argogorytes mystaceus* (Linnaeus, 1761)				1						1	early May – early July	(P) not observed (F) *Solidago* spp. flowers	En[3]	He-ot[1]
3	*Astata boops* (Schrank, 1781)		4	2	1	5				2	14	mid June – late July	(P) *Alydus calcaratus* (L.) (F) *Solidago* spp. flowers	En[3]	He-ot[1]
4	*A. kashmirensis* DD Nurse, 1909		1								1	late June – early July	(P) and (F) not observed	En[3]	He-ot[2]
5	*A. minor* Kohl, 1885		2		7	7				2	18	mid – late June	(P) and (F) not observed	En[3]	He-ot[1]
6	*Bembecinus tridens* LC (Fabricius, 1781)		17	2		10	115	59	24		227	late May – late September	(P) not observed (F) *Scleranthus* spp., *Solidago* spp. and *Thymus serpyllum* L. flowers	En[3]	He-ot[2]
7	*Bembix rostrata* LC (Linnaeus, 1758)		9	8		13		2	1		33	mid June – late August	(P) *Eristalis* spp. and *Lucilia caesar* L. (F) *Melilotus albus* Medik, *Solidago* spp., *T. serpyllum* and *Veronica spicata* L. flowers	En[3]	Dip[1]
8	*Cerceris arenaria* (Linnaeus, 1758)					2	8	7	8	10	35	mid May – late September	(P) *Brachyderes incanus* (L.) (F) *M. albus*, *Peucedanum oreoselinum* (L.) Moench, *Solidago* spp., *T. serpyllum* and *Veronica spicata* flowers	En[3]	Col[1]
**9**	*C. flavilabris* NT (Fabricius, 1793)								2		2	late July – early August	(P) not observed (F) *Solidago* spp. flowers	En[3]	Col[2]
10	*C. interrupta* (Panzer, 1799)		3			1					4	late June – late August	(P) not observed (F) *V. spicata* flowers	En[3]	Col[2]
11	*C. quadrifasciata* (Panzer, 1799)							1			1	mid June	(P) and (F) not observed	En[3]	Col[1]
12	*C. quinquefasciata* (Rossi, 1792)	3	18	1	18		2	5	12		59	mid June – mid August	(P) not observed (F) *Jasione montana* L., *P. oreoselinum* and *V. spicata* flowers	En[3]	Col[1]

13	*C. rybyensis* (Linnaeus, 1771)	2	5	23	14	14	5	7	17	8	95	mid June – late September	(P) *Halictus subauratus* (Rossi) and *H. tumulorum* (L.) (F) *Calluna vulgaris* (L.) Hull, *J. montana*, *M. albus*, *P. oreoselinum*, *Solidago* spp., *T. serpyllum* and *V. spicata* flowers	En[3]	Hym[1]
14	*Crabro cribrarius* (Linnaeus, 1758)	1	22					3			26	mid June – early August	(P) not observed (F) *P. oreoselinum*, *Solidago* spp. and *V. spicata* flowers	En(Hy) [3]	Dip[1]
15	*C. peltarius* (Schreber, 1784)		9	3			4	4			20	mid May – mid July	(P) Imagines of *Acrosathe* spp. (F) *C. vulgaris*, *P. oreoselinum*, *Solidago* spp. and *V. spicata* flowers	En[3]	Dip[1]
16	*Crossocerus annulipes* (Lepeletier de Saint Fargeau & Brullé, 1835)	3	2							1	6	late May – late August	(P) not observed (F) *Heracleum sphondylium* L., flowers and honeydew on the leaves of *Acer campestre* L. and *Ulmus minor* Mill.	Hy[3]	He-ot[1]
17	*C. capitosus* (Shuckard, 1837)	1	2								3	early September	(P) not observed (F) *P. oreoselinum* flowers	Hy[3]	Dip(He-ot)[1]
18	*C. cetratus* (Shuckard, 1837)	3									3	early August	(P) not observed (F) *H. sphondylium* flowers and honeydew on the leaves of *A. campestre*	Hy[3]	Dip[1]
19	*C. cinxius* LC (Dahlbom, 1838)		1								1	late June	(P) not observed (F) honeydew on the leaves of *Syringa vulgaris* L.	Hy[3]	Dip\He-ot[1]
20	*C. distinguendus* (A. Morawitz, 1866)		1		1						2	early – mid June	(P) not observed (F) *P. oreoselinum* flowers and honeydew on the leaves of *A. campestre*	Hy(e?) [3]	Dip[1]
21	*C. elongatulus* (Vander Linden, 1829)	1									1	mid August	(P) not observed (F) *H. sphondylium* flowers	En\Hy[3]	Dip[1]
22	*C. exiguus* (Vander Linden, 1829)	2			4	1					7	late June – late August	(P) not observed (F) honeydew on the leaves of *A. campestre* and *H. sphondylium* flowers	En[3]	He-ot[1]
23	*C. leucostoma* (Linnaeus, 1758)	1									1	late June	(P) not observed (F) *H. sphondylium* flowers	Hy[3]	Dip[1]
24	*C. megacephalus* (Rossi, 1790)	1									1	mid July	(P) not observed (F) *H. sphondylium* flowers	Hy[3]	Dip(Hym)[1]
25	*C. nigritus*	3	1								4	late June – mid August	(P) not observed (F) *P. oreoselinum* flowers	Hy[3]	Dip[1]

	Lepeletier de Saint Fargeau & Brullé, 1835														
26	*C. ovalis* Lepeletier de Saint Fargeau & Brullé, 1835				2					1	3	mid June	(P) and (F) not observed	En[3]	Dip[1]
27	*C. palmipes* (Linnaeus, 1767)			1	1					1	3	late June – mid July	(P) not observed (F) *Solidago* spp. flowers and honeydew on the leaves of *T. cordata*	En[3]	Dip[1]
28	*C. podagricus* (Vander Linden, 1829)	1			2						3	Early – Late July	(P) not observed (F) Apiaceae flowers	Hy[3]	Dip[1]
29	*C. quadrimaculatus* (Fabricius, 1793)	2	12	6	4	21			1	5	51	mid June – Late September	(P) not observed (F) Apiaceae, *Solidago* spp. flowers and honeydew on the leaves of *T. cordata*	En[3]	Dip(Tri, Lep)[1]
30	*C. styrius* DD (Kohl, 1892)	2							2		4	early July – early August	(P) not observed (F) *Solidago* spp. flowers	Hy[3]	Dip[2]
31	*C. tarsatus* LC (Shuckard, 1837)	2		1	1						4	late June – late July	(P) not observed (F) Honeydew on the leaves of *A. campestre* and *T. cordata* flowers	En[3]	Dip[1]
32	*C. vagabundus* (Panzer, 1798)		2								2	mid July – mid August	(P) not observed (F) *P. oreoselinum* flowers	Hy[3]	Dip\Lep[1]
33	*C. varus* Lepeletier de Saint Fargeau & Brullé, 1835	1				2					3	late July – late September	(P) not observed (F) Apiaceae flowers	En\Hy[3]	Dip[2]
34	*C. wesmaeli* (Vander Linden, 1829)		1	22	2	2	8	1	1		37	late May – mid September	(P) not observed (F) *H. sphondylium*, *P. oreoselinum*, *Solidago* spp. flowers and honeydew on the leaves of *T. cordata*	En[3]	Dip[1]
35	*Dinetus pictus* (Fabricius, 1793)		30	1	1	12			1		45	mid June – early September	(P) not observed (F) *J. montana*, *P. oreoselinum*, *Sedum* spp. and *V. spicata* flowers	En[3]	He-ot[1]
36	*Diodontus luperus* Shuckard, 1837		1			9					10	mid June – late September		En[3]	He-ap[1]

37	*D. minutus* (Fabricius, 1793)		34	20	3	33	9	14	1	77	191	early May – late September	(P) not observed (F) *P. oreoselinum* flowers and honeydew on the leaves of *S. vulgaris* and *T. cordata*	En[3]	He-ap[1]
38	*D. tristis* (Vander Linden, 1829)		26		1	20			1		48	mid June – late September	(P) Aphididae (F) *P. oreoselinum* flowers	En[3]	He-ap[1]
39	*Dryudella pinguis* LC (Dahlbom, 1832)							1			1	late June	(P) and (F) not observed	En[3]	He-ot[1]
40	*D. stigma* (Panzer, 1809)		10			8	3	3	3		27	mid June – mid September	(P) and (F) not observed	En[3]	He-ot[1]
41	*Ectemnius cavifrons* (Thomson, 1870)	1			1						2	mid June – mid August	(P) not observed (F) *Solidago* spp. flowers	Hy[3]	Dip[1]
42	*E. continuus* (Fabricius, 1804)	17	5	1	11		2	1	3	4	44	mid May – mid September	(P) not observed (F) *H. sphondylium*, *P. oreoselinum* and *Solidago* spp. flowers	Hy[3]	Dip[1]
43	*E. dives* (Lepeletier de Saint Fargeau & Brullé, 1835)	1	2	1							4	mid June – late July	(P) not observed (F) *P. oreoselinum* flowers	Hy[3]	Dip[1]
44	*E. fossorius* VU (Linnaeus, 1758)	1									1	early July	(P) not observed (F) *H. sphondylium* flowers	Hy[3]	Dip[1]
45	*E. guttatus* (Vander Linden, 1829)		1								1	early July	(P) and (F) not observed	Hy[3]	Dip[1]
46	*E. lapidarius* (Panzer, 1804)	4	1	1	6		2		3		17	mid May – late September	(P) not observed (F) *H. sphondylium*, *P. oreoselinum* and *Solidago* spp. flowers	Hy[3]	Dip[1]
47	*E. rubicola* (Dufour & Perris, 1840)		2								2	Mid June	(P) not observed (F) *P. oreoselinum* flowers	Hy[3]	Dip[1]
48	*Entomognathus brevis* (Vander Linden, 1829)		1		2						3	late June – early July	(P) not observed (F) *P. oreoselinum* and *Solidago* spp. flowers	En[3]	Col[1]
49	*Gorytes fallax* LC Handlirsch, 1888	3							1		4	late June – early July	(P) not observed (F) *H. sphondylium* flowers	En[3]	He-ot[2?]
50	*G. laticinctus* (Lepeletier de Saint-Fargeau, 1832)	1	1		3						5	Early July – mid August	(P) not observed (F) *H. sphondylium* and *Solidago* spp. flowers	En[3]	He-ot[1]

51	*G. quadrifasciatus* (Fabricius, 1804)				1				1		2	Mid June – early July	(P) not observed (F) Apiaceae flowers	En[3]	He-ot[1]
52	*G. quinquecinctus* (Fabricius, 1793)	6	5				1				12	late June – mid July	(P) not observed (F) *H. sphondylium* and *P. oreoselinum* flowers	En[3]	He-ot[1?]
53	*G. quinquefasciatus* (Panzer, 1798)		17	3	1		1		1		23	mid June – mid July	(P) not observed (F) *P. oreoselinum* flowers	En[3]	He-ot[2]
54	*Harpactus elegans* LC Lepeletier de Saint-Fargeau, 1832	1	3			2	2	2	11		21	early June – early July	(P) not observed (F) *Sedum* spp. flowers	En[3]	He-ot[2]
55	*H. laevis* NT (Latreille, 1792)		2						2	1	5	mid July – mid September	(P) and (F) not observed	En[3]	He-ot[2]
56	*H. lunatus* (Dahlbom, 1832)		7					1		1	9	late June – early September	(P) and (F) not observed	En[3]	He-ot[2]
57	*H. pulchellus* NT A. Costa, 1859						2	21			23	late May – late August	(P) and (F) not observed	En[1,?]	He-ot[1,?]
58	*Lestica alata* LC (Panzer, 1797)		7							16	23	mid June – early July	(P) *Deltote bankiana* (F.), *Chrysoteuchia culmella* (L.) and *Crambus lathoniellus* (Zincken) (F) *V. spicata* flowers	En[3]	Lep[1]
59	*L. clypeata* (Schreber, 1759)	2		1	11		1	11	8		34	late May – late August	(P) not observed (F) *P. oreoselinum*, *Solidago* spp. flowers and honeydew on the leaves of *Padus avium* Mill.	Hy[3]	Lep[1]
60	*L. subterranea* NT (Fabricius, 1775)	1	1				1	2		1	6	late May – mid August	(P) not observed (F) *P. oreoselinum* and *Solidago* spp. flowers	En[3]	Lep[1]
61	*Lindenius albilabris* (Fabricius, 1793)	2	4	3	5						14	late June – early September	(P) not observed (F) *P. oreoselinum* and *Solidago* spp. flowers	En[3]	He-ot\Dip[1]
62	*L. panzeri* (Vander Linden, 1829)			2	6	2	2				12	mid June – mid August	(P) not observed (F) *P. oreoselinum* and *Solidago* spp. flowers	En[3]	Dip[1]
63	*L. pygmaeus armatus* (Rossi, 1794)	2	2	8	10	21					43	mid May – late September	(P) not observed (F) *P. oreoselinum* and *Solidago* spp. flowers	En[3]	Hym(Dip)[1]

64	*Mellinus arvensis* (Linnaeus, 1758)	1	1	22	8	22		21	38	1	114	early August – late September	(P) *Helina depuncta* (Fall.), *Pollenia* spp. (F) *P. oreoselinum*, *H. sphondylium* and *Solidago* spp. flowers	En[3]	Dip[1]
65	*Mimesa bicolor* (Jurine, 1807)	3									3	early July	(P) not observed (F) *H. sphondylium* flowers	En[3]	He-ot[2]
66	*M. bruxellensis* Bondroit, 1934	2	1					1			4	early June – early July	(P) not observed (F) *H. sphondylium* flowers	En[3]	He-ot[2]
67	*M. equestris* (Fabricius, 1804)		1					6			7	mid August – mid September	(P) not observed (F) *C. vulgaris* and *P. oreoselinum* flowers	En[3]	He-ot[1]
68	*Mimumesa atratina* (F. Morawitz, 1891)	2						2			4	mid – late June	(P) not observed (F) *H. sphondylium* flowers	En[3]	He-ot[1]
69	*M. beaumonti* NT (van Lith, 1949)		1								1	mid June	(P) not observed (F) *S. vulgaris* leaves	Hy[3]	He-ot[1?]
70	*M. dahlbomi* (Wesmael, 1852)	1									1	late May	(P) not observed (F) *H. sphondylium* flowers	Hy[3]	He-ot[1]
71	*Mimumesa littoralis* EN (Bondroit, 1934)									3	3	early July – mid August	(P) and (F) not observed	En[3]	He-ot[1]
72	*Miscophus ater* DD Lepeletier de Saint-Fargeau, 1845		8	2	1	17	16	8		12	64	late May – late September	(P) and (F) not observed	En[3]	Are[2]
73	*M. bicolor* Jurine, 1807		1			14	6	4	1	3	29	mid June – late September	(P) and (F) not observed	En[3]	Are[2]
74	*M. concolor* LC Dahlbom, 1845					6	1			2	9	mid June – early	(P) and (F) not observed	En[3]	Are[1]
												August			
75	*M. niger* LC Dahlbom, 1844					5	1			2	8	late July – late August	(P) and (F) not observed	En[3]	Are[2]
76	*M. postumus* VU Bischoff, 1921						2	3			5	mid June – mid August	(P) and (F) not observed	En[3]	Are[2?]
77	*M. spurius* LC (Dahlbom, 1832)		1			3	1	4		3	12	early June – late August	(P) and (F) not observed	En[3]	Are[2?]
78	*Nysson dimidiatus* Jurine, 1807		2						2		4	early July	(P) Kleptoparasite (F) Apiaceae flowers	(En)[3]	Cle[2]
79	*N. distinguendus*					1		1			2	mid June	(P) Kleptoparasite	(En)[3]	Cle[2]

	Chevrier, 1867												(F) Apiaceae flowers		
80	*N. maculosus*<br>(Gmelin, 1790)	1	4	1	2				4		12	mid June –<br>early August	(P) Kleptoparasite<br>(F) Apiaceae flowers	(En)[3]	Cle[2]
81	*N. niger*<br>LC<br>Chevrier, 1868		1								1	early July	(P) Kleptoparasite<br>(F) not observed	(En)[3]	Cle[2]
82	*N. trimaculatus*<br>(Rossi, 1790)		2	1							3	mid July –<br>mid August	(P) Kleptoparasite<br>(F) Honeydew on the leaves of *T. cordata*	(En)[3]	Cle[2]
83	*Oxybelus argentatus*<br>LC<br>Curtis, 1833			2	1		3				6	mid June –<br>early August	(P) and (F) not observed	En[3]	Dip[2]
84	*O. bipunctatus*<br>Olivier, 1812	1	34	27	1	38	3	6	12	14	136	early June –<br>mid Septem-ber	(P) Imagines of Stratiomyi-dae, *Pollenia* spp.<br>(F) *H. sphondylium* and *P. oreoselinum* flowers	En[3]	Dip[1]
85	*O. haemorrhoidalis*<br>Olivier, 1812	1	2		3		2			2	10	mid June –<br>early August	(P) Imagines of *Sarcophaga* spp. and Tachnidae<br>(F) *P. oreoselinum* flowers	En[3]	Dip[2]
86	*O. mandibularis*<br>Dahlbom, 1845	2	7	2		1					12	mid June –<br>late July	(P) not observed<br>(F) Apiaceae flowers	En[3]	Dip[1]
87	*O. quatuordecimno-tatus*<br>Jurine, 1807		4	1	2						7	late June –<br>late July	(P) not observed<br>(F) Apiaceae flowers	En[3]	Dip[2]
88	*O. trispinosus*<br>(Fabricius, 1787)					4	1				5	mid July –<br>late August	(P) and (F) not observed	En[3]	Dip[1]
89	*O. uniglumis*<br>(Linnaeus, 1758)	2	6	14	48						70	mid June –<br>mid Septem-ber	(P) Imagines of *Anthomyia* spp.<br>(F) *P. oreoselinum* flowers	En[3]	Dip[1]
90	*O. variegatus*<br>LC<br>Wesmael, 1852	5	8	4	2		1				20	late May –<br>early August	(P) not observed<br>(F) *P. oreoselinum* flowers	En[3]	Dip[1]
91	*Passaloecus borealis*<br>Dahlbom, 1844								1		1	late August	(P) and (F) not observed	Hy[3]	He-ap[2]
92	*P. brevilabris*<br>DD<br>Wolf, 1958							2			2	late June	(P) and (F) not observed	Hy[3]	He-ap[1?]
93	*P. gracilis*<br>(Curtis, 1834)				1			2			3	late June –<br>early July	(P) and (F) not observed	Hy[3]	He-ap[1]
94	*P. pictus*<br>Ribaut, 1952	1				1		1			3	mid – late<br>July	not observed	En[4]	He-ap[2]
95	*P. singularis*<br>Dahlbom, 1844	3	3	2	1					1	10	late June –<br>mid August	(P) not observed<br>(F) Honeydew on the leaves of *T. cordata*	Hy[3]	He-ap[1]

96	*Pemphredon fabricii* M. Müller, 1911	5		10							15	mid June – late July	(P) not observed (F) *H. sphondylium* flowers and honeydew on the leaves of *T. cordata*	Hy[3]	He-ap[2?]
97	*P. inornata* Say, 1824	7	2	3					2		14	mid May – early August	(P) not observed (F) *Solidago* spp. flowers and honeydew on the leaves of *T. cordata*	Hy[3]	He-ap[1]
98	*P. lethifer* (Shuckard, 1837)	1	3			1			2	1	8	mid May – mid Septem-ber	(P) not observed (F) Apiaceae and *Solidago* spp. flowers	Hy[3]	He-ap[1]
99	*P. lugubris* (Fabricius, 1793)	1	1						3		5	mid May – mid July	(P) not observed (F) *Solidago* spp. flowers	Hy[3]	He-ap[1]
100	*P. montana* Dahlbom, 1844	1	1	1	2				1		6	late May – mid August	(P) not observed (F) Honeydew on the leaves of *T. cordata*	Hy[3]	He-ap[1]
101	*P. rugifer* (Dahlbom, 1844)		1								1	early August	(P) and (F) not observed	Hy[3]	He-ap[2]
102	*P.* spp.	4	4	1				1			10	late May – late August	(P) and (F) not observed	Hy[3]	He-ap[1]
103	*P. wesmaeli* (A. Morawitz, 1864)	1	1								2	mid June – early July	(P) and (F) not observed	Hy[3]	He-ap[1]
104	*Philanthus triangu-lum* (Fabricius, 1775)	4	16	10	3	4	1	6	3	6	53	mid June – mid Septem-ber	(P) Imagines of *Apis mellifera* L. (F) *M. albus*, *Solidago* spp., *T. serpyllum* and *V. spicata* flowers	En[3]	Hym[1]
105	*Psenulus laevigatus* (Schenck, 1857)	2		3	1						6	mid May – mid July	(F) Honeydew on the leaves of *T. cordata*	Hy[3]	He-ap[2]
106	*P. meridionalis* de Beaumont, 1937				1						1	early July	(P) not observed (F) *Solidago* spp. flowers	Hy[3]	He-ap[2?]
107	*P. pallipes* (Panzer, 1798)	6	1	7	1						15	late May – mid August	(P) not observed (F) *Solidago* spp. flowers and honeydew on the leaves of *T. cordata*	Hy[3]	He-ap[1]
108	*P. schencki* (Tournier, 1889)	1			1						2	mid May – late June	(P) and (F) not observed	Hy[3]	He-ap[1]
109	*Rhopalum coarcta-tum* (Scopoli, 1763)			5							5	mid July – early Sep-tember	(P) and (F) not observed	Hy[3]	Dip(Pso,Neu)[1]
110	*Solierella compedita* (Piccioli, 1869)					1					1	mid June	(P) and (F) not observed	Hy(En)[3]	He-ot[2]
111	*Spilomena enslini* Blüthgen, 1953		1								1	early August	(P) and (F) not observed	Hy[3]	Thy[1]
112	*Stigmus solskyi*				1						1	late June	(P) and (F) not observed	Hy[3]	He-ap[1]

	A. Morawitz, 1864														
113	*Stizus perrisi*<br>CR<br>Dufour, 1838						4	2			6	late May – late	(P) not observed<br>(F) *P. oreoselinum* flowers	En[3]	Ort[2]
												June			
114	*Tachysphex austria-cus*<br>Kohl, 1892		1	1			1	4			7	early June – late June	(P) and (F) not observed	En[3]	Ort[1?]
115	*T. fulvitarsis*<br>LC<br>(A. Costa, 1867)		3	1	1	1	1	1	1		9	mid June – mid July	(P) not observed<br>(F) *Sedum* spp. flowers	En[3]	Ort[1]
116	*T. helveticus*<br>Kohl, 1885		5		1	7	4			5	22	late May – mid August	(P) not observed<br>(F) *P. oreoselinum*, *Scleran-thus* spp. and *T. serpyllum* flowers	En[3]	Ort[1]
117	*T. nitidus*<br>(Spinola, 1806)					1	18	7	1		27	late April – early August	(P) not observed<br>(F) *Scleranthus* spp., *Solidago* spp. and *T. serpyllum* flowers	En[3]	Ort[1]
118	*T. obscuripennis*<br>(Schenck, 1857)	2	1			22	22	9	4	27	87	early June – mid August	(P) not observed<br>(F) *P. oreoselinum*, *Solidago* spp. and *T. serpyllum* flowers	En[3]	Bla[1]
119	*T. panzeri*<br>(Vander Linden, 1829)		1	1		4	12	22	1		41	early June – late July	(P) not observed<br>(F) *P. oreoselinum*, *Solidago* spp. and *T. serpyllum* flowers	En[3]	Ort[2]
120	*T. pompiliformis*<br>(Panzer, 1803)		17	6		7		5	1	13	49	early May – mid August	(P) not observed<br>(F) *P. oreoselinum*, *Solidago* spp. and *T. serpyllum* flowers	En[3]	Ort[1]
121	*T. psammobius*<br>LC<br>(Kohl, 1880)		11	1	1		1	2	1	2	19	late April – mid July	(P) not observed<br>(F) *P. oreoselinum* and *T. ser-pyllum* flowers	En[3]	Ort[2?]
122	*T. tarsinus*<br>DD<br>(Lepeletier de Saint-Fargeau, 1845)						2	2		1	5	late June – early July	(P) and (F) not observed	En[3]	Ort[2?]
123	*T. unicolor*<br>(Panzer, 1809)		3			1		3	5	3	15	early June – mid August	(P) not observed<br>(F) *T. serpyllum* flowers	En[3]	Ort[2]
124	*Trypoxylon attenua-tum*<br>F. Smith, 1851	1		6							7	late June – late July	(P) not observed<br>(F) Honeydew on the leaves of *T. cordata*	Hy[3]	Are[1]
125	*T. clavicerum*<br>Lepeletier de Saint Fargeau and Audinet-Serville, 1828	3	3								6	early June – late July	(P) not observed<br>(F) Honeydew on the leaves of *T. cordata*	Hy[3]	Are[1]
126	*T. deceptorium*<br>Antropov, 1991	1		2	1	1					5	mid July – late August	(P) not observed	Hy[3]	Are[2?]

													(F) Honeydew on the leaves of *T. cordata*		
127	*T. figulus* (Linnaeus, 1758)		2		1						3	mid June – late August	(P) not observed (F) *S. vulgaris* leaves	Hy[3]	Are[1]
128	*T. medium* de Beaumont, 1945	1								1	2	mid June – early August	(P) and (F) not observed	Hy[3]	Are[2]
129	*T. minus* de Beaumont, 1945	15	7	16	6	1		8	4	1	58	late May – late August	(P) not observed (F) Honeydew on the leaves of *T. cordata*	Hy[3]	Are[2?]
130	*Ammophila cam-pestris* Latreille, 1809			1		7	45	30	1	3	87	may – late August	(P) not observed (F) *T. serpyllum* and *Solidago* spp. flowers	En[3]	Hym[1]
131	*A. pubescens* Curtis, 1836					3				5	8	late May – mid August	(P) Larvae of Geometridae (F) *Solidago* spp. flowers	En[3]	Lep(Hym)[1]
132	*A. sabulosa* (Linnaeus, 1758)		76	15	8	29	14	21	21	68	252	mid May – early Octo-ber	(P) Larvae of *Panolis flammea* (Denis & Schiff.) (F) *J. montana*, *Soli-dago* spp., *Teucrium* spp., *T. serpyllum* and *V. spicata* flowers	En[3]	Lep[1]
133	*Podalonia affinis* (W. Kirby, 1798)		17		1	11	3	3	2		37	mid May – mid Septem-ber	(P) not observed (F) *V. spicata* and *P. oreoselinum* flowers	En[3]	Lep[1]
134	*P. hirsuta* (Scopoli, 1763)			1		7			7	13	28	late May – mid Septem-ber	(P) not observed (F) *P. oreoselinum*, *Solidago* spp. and *V. spicata* flowers	En[3]	Lep[1]
135	*P. luffii* (Saunders, 1903)						1	15	1		17	late May – mid Septem-ber	(P) not observed (F) *T. serpyllum* flow-ers	En[3]	Lep[2]
136	*Sphex funerarius* Gussakovskij, 1934			1			5	27			33	mid July – early Sep-tember	(P) not observed (F) *T. serpyllum* and *M. albus* flowers	En[3]	Ort[2]
	**Total of specimens (N)**	152	563	312	220	409	339	375	224	322	**2916**				
	**Number of species (S)**	59	81	52	53	48	43	50	46	38	**136**				

[1] Lomholdt 1984; [2] Blösch 2000; [3] Witt 2009; [4] Janvier 1961; Abbreviations: Status: CR - critically endangered, EN – Endangered , VU – vulnerable, NT - near threatened, LC - least concern, DD - data-deficient, Nesting: En – endogeic (digging nests in the soil and occupying existing cracks in the soil), Hy - hypogeic (nesting above the ground in woods or plant shoots.), Prey: Dip – Diptera, Col – Coleoptera, Hym – Hymenoptera, Lep – Lepidoptera, Tri – Trichoptera, Ort – Orthoptera, Are – Araneae, Bla – Blattodea, He-ap – Hemiptera-Aphidoidea, He-ot – Hemiptera-other, Thy – Thysanoptera, Pso – Psocoptera, Neu – Neuroptera.

Table s2. Flight periods for the 15 most abundant species (those with 50+ records, shown in dscending order of abundance). Months are represented by their numerals, 5=May etc.

Species	n		5			6			7			8			9			10	
Ammophila sabulosa	252		■	■	■	■	■	■	■	■	■	■	■	■	■	■	■		
Bembecinus tridens	227			■	■	■	■	■	■	■	■	■	■	■	■	■			
Diodontus minutus	191	■	■	■	■	■	■	■	■	■	■	■	■	■	■	■			
Oxybelus bipunctatus	136				■	■	■	■	■	■	■	■	■	■	■				
Mellinus arvensis	114										■	■	■	■	■	■			
Cerceris rybyensis	95					■	■	■	■	■	■	■	■	■	■	■			
Tachysphex obscuripennis	87				■	■	■	■	■	■	■	■							
Ammophila campestris	87	■	■	■	■	■	■	■	■	■	■	■	■						
Oxybelus uniglumis	70					■	■	■	■	■	■	■	■	■	■				
Miscophus ater	64			■	■	■	■	■	■	■	■	■	■	■	■	■			
Alysson spinosus	62				■	■	■	■	■	■	■								
Cerceris quinquefasciata	59					■	■	■	■	■	■	■							
Trypoxylon minus	58			■	■	■	■	■	■	■	■	■	■						
Philanthus triangulum	53					■	■	■	■	■	■	■	■	■	■				
Crossocerus quadrimaculatus	51					■	■	■	■	■	■	■	■	■	■	■			
